# Bio-Based Gum Arabic-Reinforced Epoxy Overlay System: Mechanical, Thermal, and Tribological Performance with Wear Mechanism Analysis

**DOI:** 10.3390/polym18141695

**Published:** 2026-07-09

**Authors:** Amirthalakshmi Alavanthar, Shubrajit Bhaumik, Megha Sasidharan Nisha, Kiran Mangalampalli, Viorel Paleu, Vitalie Florea

**Affiliations:** 1 Department of Science and Humanities, Amrita School of Engineering, Amrita Vishwa Vidyapeetham, Chennai 601103, Tamil Nadu, India; a_amirthalakshmi@ch.students.amrita.edu; 2 Tribology and Interactive Surfaces Research Laboratory (TRISUL), Department of Mechanical Engineering, Amrita School of Engineering, Amrita Vishwa Vidyapeetham, Chennai 601103, Tamil Nadu, India; 3 Department of Physics and Nanotechnology, SRM Institute of Science and Technology, Kattankulathur 603203, Tamil Nadu, India; megha.s.n2244@gmail.com; 4 Centre for Interdisciplinary Research, SRM University-AP, Amravati 522240, Andhra Pradesh, India; kiran.k@srmap.edu.in; 5 Department of Mechanical Engineering, Mechatronics and Robotics, “Gheorghe Asachi” Technical University of Iași, 43 Prof. D. Mangeron Blvd, 700050 Iasi, Romania; 6 Department of Structural Mechanics, “Gheorghe Asachi” Technical University of Iași, 1 Prof. Dimitrie Man-geron Blvd, 700050 Iasi, Romania

**Keywords:** epoxy, gum arabic, overlays, thick coatings, surface wear, TOPSIS optimisation, nanoindentation, adhesion strength

## Abstract

This study investigates the tribological performance of gum arabic (GA)-reinforced epoxy (EP) overlays on EN8 steel. Four GA concentrations (0.25, 0.5, 1, and 3 wt.%) were incorporated into the epoxy matrix to prepare overlays designated as EPGA1–EPGA4. Tribological performance was evaluated using a reciprocating tribometer under varying loads (5–20 N), sliding frequencies (1–2.5 Hz), and temperatures (40–70 °C). An L16 orthogonal array based on the Taguchi method was used to design the experimental matrix, and multi-criteria decision-making using the TOPSIS technique was employed to identify the optimum tribological condition based on minimum coefficient of friction (COF) and specific wear rate (SPWR). The optimum condition was obtained for the EPGA3 overlay (1 wt.% GA) at 5 N, 2 Hz, and 60 °C, which exhibited the lowest COF of 0.0567 ± 0.0021 and negligible wear. In contrast, the pure epoxy overlay showed severe adhesive wear, catastrophic delamination, a high COF of 1.15 ± 0.0023, and a wear rate of 163 × 10^−8^ mm^3^/Nm. Thermal characterization showed that GA improved the thermal stability and thermal transition behaviour of the epoxy matrix. Thermogravimetric analysis revealed an increase in onset degradation temperature from 320 °C for pure EP to 342 °C for EPGA4, while differential scanning calorimetry showed that EPGA3 exhibited the highest glass transition temperature (~118 °C), indicating improved interfacial interactions and restricted polymer-chain mobility. Nanoindentation and pull-off adhesion tests further confirmed the improved mechanical integrity and interfacial adhesion of the GA-reinforced overlays, demonstrating its potential as a sustainable reinforcement for tribological coating applications.

## 1. Introduction

The development of advanced coating materials has become a major research focus in tribology due to the increasing demand for reducing friction, wear, and surface degradation in engineering systems. Steel is widely used in structural and mechanical components; however, its surfaces often require protective coatings to improve durability and performance under harsh operating conditions such as sliding contact, temperature variation, and mechanical loading [1]. Among the available protective materials, epoxy resin coatings have attracted considerable attention because of their excellent adhesion to metallic substrates, high mechanical strength, chemical resistance, electrical insulation, and low curing shrinkage [2,3,4,5,6,7,8,9]. Owing to these advantages, epoxy coatings are widely used in applications such as aerospace structures, cryogenic storage tanks, marine components, protective coatings, adhesives, and electric vehicle systems [10]. Despite these beneficial properties, pure epoxy coatings suffer from inherent limitations such as brittleness, relatively high coefficient of friction (COF), and limited wear resistance under sliding conditions [11]. During tribological contact, brittle fracture, crack initiation, and coating delamination often accelerate material removal, thereby reducing the service life of epoxy coatings. Consequently, considerable research has focused on incorporating reinforcing fillers into epoxy matrices to improve their load-bearing capacity, fracture toughness, and wear resistance [12]. While inorganic fillers such as graphene, boron nitride, zirconia, silica, and titanium dioxide have demonstrated significant improvements in tribological performance, their high cost, processing complexity, agglomeration tendency, and environmental concerns have motivated researchers to explore sustainable alternatives. To overcome these limitations, researchers have introduced various fillers and reinforcements into epoxy matrices to enhance their mechanical and tribological properties. For example, Kurahatti et al. [13] incorporated zirconia (ZrO_2_) nanoparticles (0.5–10 wt.%) into LY556 epoxy and reported improved wear resistance due to the rolling effect of nanoparticles between sliding surfaces. Similarly, Ghorbani et al. [14] introduced TiO_2_ nanoparticles and linseed oil microcapsules into epoxy coatings, resulting in enhanced self-lubrication and reduced debris formation. Huang et al. [15] developed epoxy composite coatings containing cubic boron nitride (CBN) particles, where micro-sized particles improved load-bearing capacity and nano-sized particles enhanced toughness. Although these inorganic fillers significantly improve tribological performance, their high cost, processing complexity, and environmental concerns have encouraged researchers to explore alternative sustainable reinforcements. In recent years, bio-based materials have gained increasing attention as environmentally friendly reinforcements for polymer composites [16]. Natural materials derived from plant sources offer several advantages, including biodegradability, low density, renewability, and cost-effectiveness [17,18,19,20]. Various natural fibers such as kenaf, jute, hemp, sisal, ramie, banana, and pineapple leaf fibers have been studied for reinforcing polymer matrices due to their favorable mechanical properties and sustainable nature [21,22,23]. In addition, agricultural waste materials such as corncob particles [24] and pistachio shell powders [25] have also been incorporated into epoxy matrices to improve wear resistance and mechanical performance. Although epoxy coatings are widely used because of their excellent adhesion, corrosion resistance, chemical stability, and ease of processing, their tribological performance is often limited by inherent brittleness, susceptibility to crack initiation and propagation, and poor resistance to coating delamination under repeated sliding conditions. These limitations can lead to unstable friction behaviour and accelerated material removal, particularly under elevated load and temperature. To overcome these drawbacks, different inorganic fillers have been incorporated into epoxy matrices to improve hardness, toughness, and wear resistance [26]. However, the use of such fillers is often associated with high cost, agglomeration tendency, and limited sustainability [27]. In this context, gum arabic (GA) represents a promising bio-based reinforcement because of its natural film-forming ability, high surface activity, and abundance of hydroxyl and carboxyl functional groups. These functionalities can promote hydrogen-bonding interactions with the epoxy network, improve filler–matrix interfacial adhesion, and facilitate more efficient stress transfer within the coating. In addition, the film-forming nature of GA is expected to support the formation of a stable protective tribo-layer during sliding, thereby reducing direct asperity contact, crack initiation, and material removal. Therefore, GA can be considered a sustainable and promising reinforcement for improving the tribological performance of epoxy coatings.

In particular, GA, a natural polysaccharide obtained from the sap of Acacia trees, has recently attracted attention as a potential bio-based additive for polymer systems [28]. GA primarily consists of complex polysaccharides containing sugars such as galactose, arabinose, rhamnose, glucose, and glucuronic acid [29,30]. Owing to its natural film-forming ability, surface activity, and excellent compatibility with various materials, GA has been widely used in pharmaceutical, food, and cosmetic industries as a stabiliser, emulsifier, and binder [31,32,33]. These functional characteristics suggest that GA may also play a beneficial role in modifying polymer coatings by improving interfacial bonding and promoting the formation of protective films during sliding [34,35]. Despite its promising properties, the application of gum arabic as a reinforcement in epoxy tribological coatings has not been systematically explored. In particular, its potential role in reducing friction, enhancing wear resistance, and improving coating durability under sliding conditions remains largely unexplored. Therefore, incorporating GA into epoxy coatings may provide a sustainable strategy to overcome the limitations of conventional epoxy systems while simultaneously improving tribological performance.

In this context, the present study investigates the effect of gum arabic incorporation on the tribological behaviour of epoxy composite coatings applied on EN8 steel substrates. The coatings were evaluated under different loads, sliding frequencies, and temperatures using a reciprocating tribometer. Additionally, mechanical properties were assessed through nanoindentation testing, while coating adhesion was evaluated using pull-off strength measurements. Thermal behaviour was examined using TGA analyses, and surface morphology of worn coatings was analysed using scanning electron microscopy. The objective of this work is to develop a bio-based epoxy composite coating with improved friction and wear performance while maintaining strong adhesion and mechanical durability. The findings of this study provide new insights into the use of natural biopolymers for designing sustainable, high-performance tribological coatings for steel surfaces operating under moderate sliding conditions.

## 2. Materials and Methods

### 2.1. Selection of Material

In this study, Araldite LY556 epoxy resin and Aradur HY 951 hardener were sourced from Fiber Region, Chennai, India. GA powders, steel bars (EN8 grade, 50 × 20 mm^2^, hardness: 15 ± 1 HRC), and steel balls (EN31 grade, Ø12.7 mm, hardness: 55 ± 0.55 HRC, average surface roughness: 0.1107 ± 0.0026 µm) were also sourced Fiber Region, Chennai, India. Table 1 shows the composition of EN8 and EN31 steels.

### 2.2. Surface Preparation of the Steel Bars for Coating

Zhang S et al. [36] reported that mechanical treatment methods are a powerful means to achieve precise control over the roughness of substrate surfaces. Sandpaper grinding (Grade 24 grit) as a mechanical treatment method was used in this work to polish steel bars, achieving an average surface roughness of 3.461 ± 0.417 µm. The surface roughness was measured using a 2D profilometer (Make: Zeiss (Oberkochen, Germany), Model: Surfcom Touch 50).

### 2.3. Preparing the EP/EPGA Coated Steel Bars

**Figure 1:**
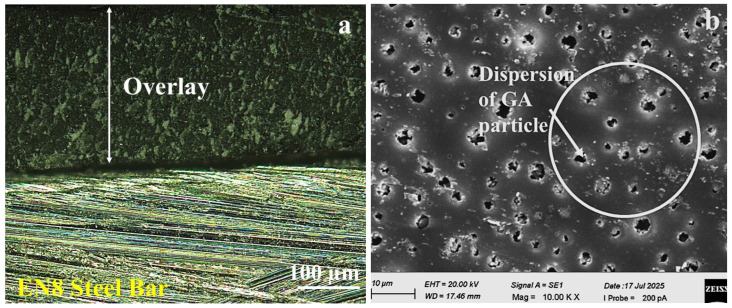
(**a**) Optical microscope image of EP overlay on EN8 steel bars (**b**) SEM Dispersion of EP overlay reinforced with GA particles.

GA was incorporated into the resin at varying weight percentages (wt.%): EP (0 wt.%), EPGA1 (0.25 wt.%), EPGA2 (0.50 wt.%), EPGA3 (1 wt.%), and EPGA4 (3 wt.%). To enhance the processability of the EP, it was blended with 10 wt.% of 2-ethoxyethanol and 10 wt.% of toluene. The curing agent HY951 was added in a 100:10 ratio (EP to hardener) and mechanically stirred for 40 min to ensure complete mixing. For the composite formulation, the required quantity of GA powder was first mixed with 2-ethoxyethanol and manually stirred using a glass rod for 15 min, which enabled the formation of a stable dispersion. This pre-dispersed GA solution was then added to the EP/hardener/toluene mixture and stirred thoroughly to form a homogeneous blend. The prepared coating was applied to EN8 steel bars using a double-dip method, with each dip lasting for 5 s followed by a 3 s draining period to remove excess material. Curing of the coated specimen was done in two stages: an initial 72 h air-drying at room temperature, followed by thermal curing in a muffle furnace (make: Hasthas (Chennai, India), model: HIS-115) at 60 °C for 2 h. After curing, the average coating thickness measured using an optical microscope (make: Olympus (Tokyo, Japan), model: BX53M) was 496.34 ± 10.12 µm (Figure 1a). Unlike thin hard coatings (e.g., PVD/CVD films), which typically operate in the micrometer range, polymer composite coatings function as compliant, load-distributing layers and are therefore applied at higher thicknesses. This distinction is particularly important in applications involving dry sliding or boundary lubrication, where energy dissipation and deformation accommodation play a dominant role in wear resistance.

Qasim et al. [37] reported that thick coatings (1000 µm) reduce deflections, decreasing the stresses below the coatings while the thin coatings (160 µm) experience high deflection. Earlier reported work has indicated that a relatively thick coating was beneficial for resisting crack propagation and mechanical wear [38]. Choudhury et al. [39] reported that coating thickness plays a critical role in tribological performance, with thicker coatings exhibiting longer wear life due to delayed substrate exposure and improved load-bearing capacity. Polanec et al. [40] demonstrated that very thin coatings (less than 500 nm) are rapidly removed during sliding due to insufficient thickness and adhesion, highlighting the necessity of thicker or multilayer coatings for improved durability in tribological applications. Therefore, the relatively large coating thickness employed in the present study aligns with the functional requirements of heavy-duty tribological systems rather than conventional thin-film coating applications. The proposed coating behaves more like a polymeric overlay layer rather than a conventional coating. The average surface roughness of the epoxy overlay surfaces was found to be 0.0612 ± 0.0234 µm. Figure 1b shows the scanning electron microscope images of the EP overlay reinforced with GA particles.

### 2.4. Investigating the Anti-Wear Properties of the Epoxy Overlays on Steel Surfaces

The anti-wear properties of the steel surfaces with overlays were examined using a reciprocating wear testing rig (make: Magnum Engineers, Bengaluru, India; model No. RCP01). The tests were conducted under applied normal loads of 5, 10, 15, and 20 N, reciprocating frequencies of 1, 1.5, 2, and 2.5 Hz, and temperatures of 40, 50, 60, and 70 °C at a constant stroke length of 15.1 mm. The selected experimental parameters were adopted based on the authors’ previously reported investigation of epoxy-coated steel surfaces under reciprocating sliding conditions [38], in which these operating ranges were shown to effectively capture the transition from mild wear to severe coating degradation without causing immediate catastrophic failure. Furthermore, the selected parameter ranges are consistent with the operating limits of the epoxy coating established in the previous study and therefore provide a reliable basis for evaluating the influence of GA reinforcement. The applied load range (5–20 N) enabled a systematic evaluation of the load-bearing capability of the coatings, whereas the selected frequencies (1–2.5 Hz) facilitated investigation of the influence of sliding conditions on friction and wear behaviour. The temperature range (40–70 °C) was selected to evaluate the coating performance below and approaching the glass transition temperature of the epoxy matrix, where significant changes in its mechanical and tribological behaviour are expected. The GA concentrations (0.25–3 wt.%) were selected to identify the optimum reinforcement level while assessing the influence of filler loading on particle dispersion, interfacial bonding, and tribological performance. Lower GA contents were expected to provide effective reinforcement through improved dispersion, whereas higher concentrations were included to evaluate the effects of possible particle agglomeration and the associated reduction in reinforcement efficiency. These parameter ranges therefore enabled a systematic assessment of the combined influence of mechanical loading, sliding conditions, operating temperature, and GA content on the tribological performance of the developed epoxy overlays. The experiments (Exp) were planned as per the Taguchi design of experiments (DOE). The Taguchi design of experiments (DOE) provides an efficient statistical approach for generating balanced experimental matrices while substantially reducing the number of experimental trials compared with a full factorial design [41]. In the present study, a Taguchi L16 (4^4^) orthogonal array was employed solely to generate the experimental matrix for four control factors, GA content, applied load, reciprocating frequency, and temperature, each investigated at four levels. A full factorial design would require 4^4^ (256) experimental combinations, whereas the L16 orthogonal array reduced the required number of experiments to 16 while maintaining an orthogonal and balanced distribution of factor levels. The generated experimental combinations are presented in Table 2. Minitab 18 software (version Minitab 18.1) was used to generate the L16 orthogonal array. Each test was repeated thrice, and the average of the three values is reported in this work.

### 2.5. Fourier Transform Infrared Spectroscopy of EP Overlays

**Figure 2:**
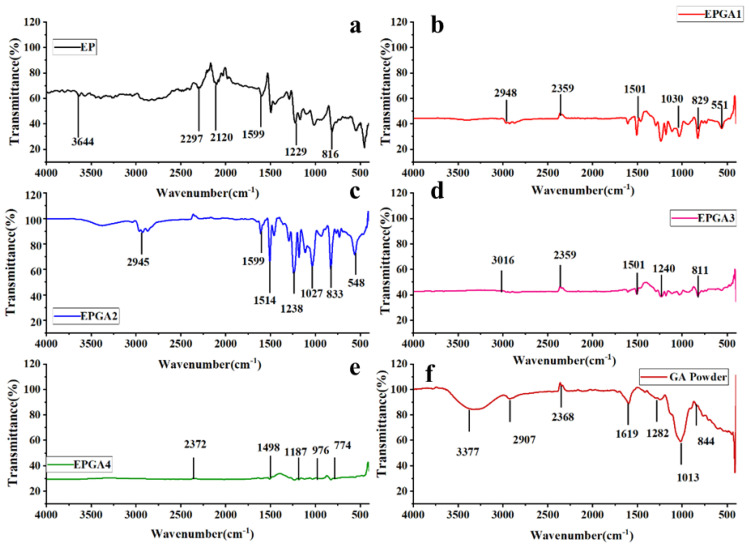
(**a**–**f**) FTIR spectral analysis of EP and EPGA overlays.

Fourier Transform Infrared Spectroscopy (FTIR) was employed to identify the functional groups present in pure epoxy (EP), gum arabic (GA) powder, and GA-incorporated epoxy overlays (EPGA1–EPGA4). The analysis was carried out using an FTIR spectrometer (Shimadzu, IR Tracer-100, Japan) with a resolution of 2 cm^−1^ over a wavenumber range of 4000–400 cm^−1^. The corresponding spectra are presented in Figure 2. The FTIR spectrum of pure EP (Figure 2a) exhibits characteristic absorption peaks associated with its molecular structure. A prominent peak observed around 915 cm^−1^ corresponds to the epoxide ring deformation, confirming the presence of reactive epoxy groups [42]. Peaks in the range of 1500–1600 cm^−1^ are attributed to aromatic C=C stretching vibrations of the bisphenol-A backbone [43]. Additionally, a broad absorption band around 3400 cm^−1^ is assigned to O–H stretching vibrations, which may arise from hydroxyl groups formed during curing or due to moisture absorption [44].

The FTIR spectrum of GA powder (Figure 2f) shows typical features of polysaccharide-based materials. The broad band in the region of 3300–3400 cm^−1^ corresponds to O–H stretching vibrations, indicating the presence of abundant hydroxyl groups [45]. Peaks observed between 1000–1150 cm^−1^ are associated with C–O–C and C–O stretching vibrations of carbohydrate structures. A band near 1600–1650 cm^−1^ is attributed to C=O stretching of uronic acid groups or associated protein fractions present in GA [46]. Furthermore, peaks in the range of 500–600 cm^−1^ correspond to skeletal vibrations and ring deformations of the polysaccharide backbone, confirming the structural integrity of GA. These observations are consistent with previously reported FTIR spectra of gum arabic [47,48].

In the composite spectra (EPGA1–EPGA4), most of the key peaks from both EP and GA were retained, confirming the physical blending and chemical compatibility of the two phases. A noticeable reduction in the intensity of the epoxide peak at 915 cm^−1^ was observed in GA-loaded samples, especially in EPGA3 and EPGA4, suggesting interaction or partial ring opening due to GA addition. Additionally, the broader O–H bands in GA-rich formulations suggest hydrogen bonding or enhanced hydrophilicity. The retention of major EP and GA peaks, along with subtle shifts or broadening, supports the formation of a physically bonded composite overlay in which GA is well dispersed in the EP matrix. This molecular interaction will influence the thermal, mechanical, and tribological behaviour of the final overlay system.

### 2.6. Determining Thermal Degradation of EP Overlays Using Thermogravimetric Analysis

**Figure 3:**
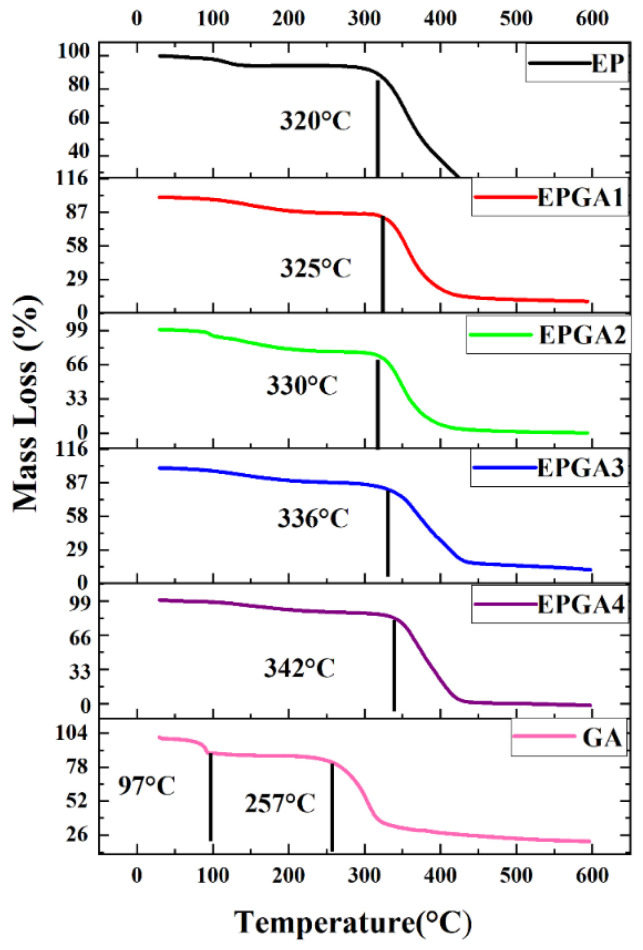
Thermogravimetric analysis of EPGA overlays.

A thermogravimetric analyser (make: PerkinElmer (Shelton, CT, USA), model: TGA8000) was used to investigate the thermal degradation behaviour and stability of the EP overlays. Figure 3 presents the mass loss curves that represent the percentage of material loss as a function of temperature under heating, revealing multiple stages of degradation [49]. The thermogravimetric analysis (TGA) data for GA, EP, and the EPGA overlays are shown in Table 3. The GA powder exhibited a multi-stage degradation profile, with an initial mass loss at 97 °C due to the evaporation of absorbed moisture a characteristic of its hygroscopic polysaccharide nature. A second major degradation stage for GA was observed at 257 °C. Vuillemin et al. [50] reported that during the early phase below 100 °C, approximately 10% of the total mass of gums were lost due to water evaporation. For the overlays, the pure EP exhibited an onset degradation temperature of 320 °C. Interestingly, the incorporation of GA led to a progressive increase in thermal stability. The onset of thermal degradation shifted to higher temperatures, reaching 336 °C for EPGA3 (1 wt.%) and 342 °C for EPGA4 (3 wt.%). This enhancement is attributed to the strong interfacial hydrogen bonding between the GA hydroxyl groups and the epoxy matrix, which restricts the mobility of the polymer chains and requires higher energy for thermal decomposition. In contrast, the GA powder left behind a char residue of about 20%, attributed to its polysaccharide-based structure and inherent carbon-rich content [51].

Furthermore, the final char residue at 600 °C increased significantly from approximately 6% in pure EP to nearly 20% in EPGA4. The elevated char yield acts as a protective carbonaceous barrier, suppressing the release of volatile products and limiting heat transfer to the underlying matrix [52,53]. These results confirm that GA not only acts as a bio-based reinforcement but also significantly enhances the thermal resistance of the epoxy system.

### 2.7. Determining the Glass Transition Temperature of the Composite Coatings Using Differential Scanning Calorimeter

**Figure 4:**
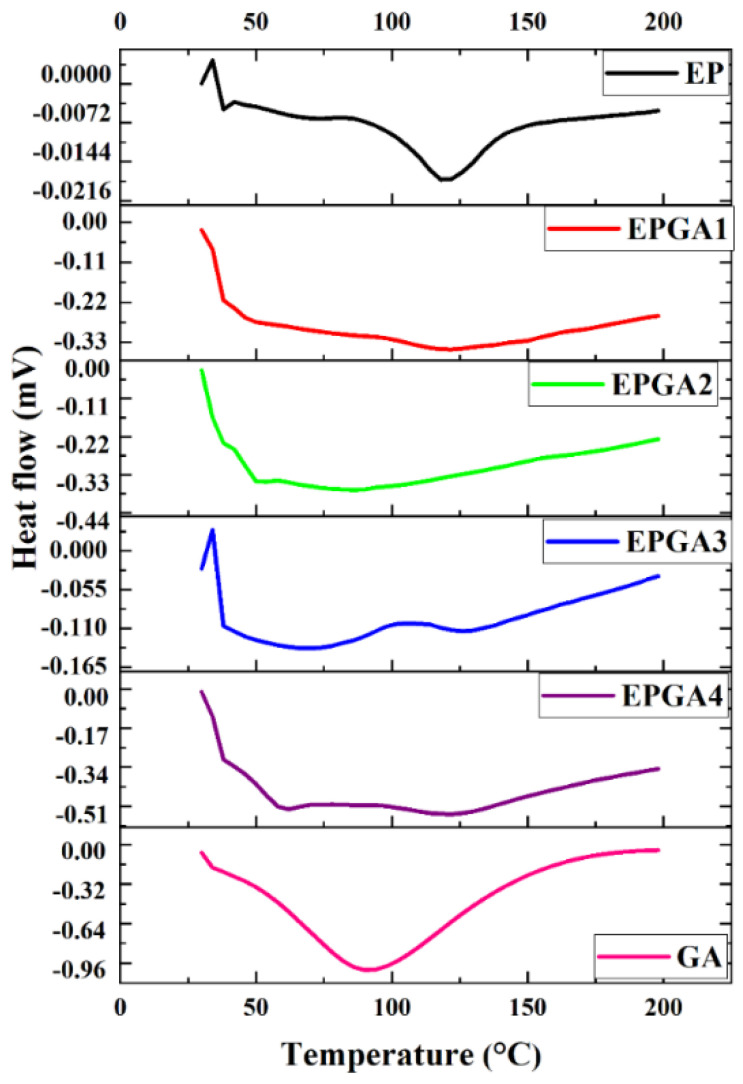
Differential scanning calorimeter analysis of EPGA overlays.

A differential scanning calorimeter (make: TA Instruments (New Castle, DE, USA), model: DSC 25) was employed to investigate the thermal transitions of the developed epoxy (EP) and gum arabic (GA)-reinforced epoxy composite coatings, with particular emphasis on the glass transition temperature (Tg). The DSC thermograms presented in Figure 4 illustrate the heat-flow response of each sample as a function of temperature, while the estimated Tg values are summarized in Table 4. All measurements were carried out under a nitrogen atmosphere at a heating rate of 10 °C min^−1^. In the thermograms, the x-axis represents temperature (°C), whereas the y-axis denotes heat flow (mV), with downward deflections corresponding to endothermic events associated with heat absorption by the sample.

The glass transition represents the transformation of the polymer from a rigid glassy state to a more flexible rubbery state, accompanied by increased molecular chain mobility. The pure EP coating exhibited a Tg of approximately 115 °C, reflecting the relatively rigid nature of the cured epoxy network. In contrast, GA powder exhibited a Tg of approximately 87 °C, which is characteristic of polysaccharide-based materials with greater molecular flexibility and moisture sensitivity. Most DSC thermograms also exhibited an initial broad endothermic region between 63 and 134 °C, which is attributed to the evaporation of physically absorbed moisture. Such moisture-loss transitions are typical of hydrophilic polymers containing abundant hydroxyl functional groups and have been similarly reported by Hirwani et al. [53].

The incorporation of GA noticeably altered the thermal transition behaviour of the epoxy matrix. The Tg values of EPGA1 and EPGA2 increased to approximately 100 °C and 105 °C, respectively, indicating restricted polymer-chain mobility arising from physical interactions, particularly hydrogen bonding, between the hydroxyl groups of GA and the epoxy matrix. Among all compositions, EPGA3 (1 wt.% GA) exhibited the highest Tg of approximately 118 °C, indicating the most effective restriction of segmental chain motion. This behaviour suggests improved interfacial interactions and more uniform dispersion of GA within the epoxy matrix, which enhances the thermal stability of the composite. However, a further increase in GA content to 3 wt.% (EPGA4) reduced the Tg to approximately 110 °C. This reduction is likely associated with particle agglomeration at higher filler loading, resulting in less efficient interfacial reinforcement and the formation of localized free volume that facilitates chain mobility.

Overall, the DSC results demonstrate that GA significantly influences the thermal transition behaviour of the epoxy matrix. An optimum GA loading of 1 wt.% provides the greatest restriction of polymer-chain mobility and the highest glass transition temperature, whereas excessive GA loading diminishes these benefits because of agglomeration-related effects.

### 2.8. Evaluating the Adhesive Strength of EP Overlays on the Steel Surface

The adhesive strength of the EP and GA-modified EP overlays was assessed using the pull-off method outlined in ASTM D 4541 [54]. This method evaluates the adhesion strength of overlays on EN8 steel substrates. In the pull-off test, a loading apparatus (often referred to as a dolly or stud, Φ20 mm) is firmly affixed perpendicularly to the surface of the overlays using Araldite adhesive. After permitting sufficient time for the adhesive to cure, the testing equipment (make: Biuged (Guangzhou, China), model: BGD500\5001905033) was attached to the fixture and accurately aligned to exert an upward tensile force perpendicular to the surface with overlays. The applied stress was progressively increased until the overlay detached from the substrate. The force necessary for separation is recorded as the adhesive strength of the overlay [55]. The pull-off test results indicated that the EP overlay exhibited an average adhesion strength of 10.34 ± 0.14 MPa (Table 5), reflecting the normal mechanical interlocking and chemical bonding that EP exhibits with steel. The incorporation of GA in the EP matrix resulted in a significant increase in bonding strength, which is almost double that of the EP overlay. However, all GA-incorporated overlays exhibited similar adhesion strength.

The enhanced adhesion can be linked to the function of GA in strengthening the interfacial bonding between the EP matrix and the steel surface. The hydrophilic characteristics of GA, combined with its ability to form films, probably enhanced surface wetting and mechanical interlocking. It was also reported by Alhassan et al. [56] that the chemical modification of GA improves its film-forming capability and mechanical strength, leading to better reinforcement and interfacial bonding in composite materials.

### 2.9. Evaluating the Mechanical Properties Using Nano-Indentation of the Overlay

The mechanical characteristics of the proposed EP and GA-based EP overlays were assessed using a nanoindenter. A sharp diamond Berkovich-type indenter was applied to the sample’s surface under regulated stress [57]. All indentation experiments were carried out at room temperature using a NIOS advanced nano hardness tester (make: Ostec Instruments, Moscow, Russia) with a maximum load of 1.25 N. To determine the material’s deformation resistance, the load-displacement data acquired from the equipment were analysed. To reduce substrate impacts, particular emphasis was placed on ensuring that the deepest indentation did not exceed 10% of the total overlay thickness. The load vs. depth curves were examined utilizing the software ‘NanoScan Device Control Software Version 202.892’ supplied by the supplier to determine the hardness and elastic moduli. The indentation experimental values were derived using the equations from the Oliver and Pharr technique [58]. The indentation hardness was calculated using Equation (1).

(1) $$H=\frac{P_{max}}{A}$$

where H is the hardness, Pmax denotes the maximum normal load, and A represents the projected contact area at this maximum load. Young’s modulus of the EP composites was determined using Equation (2).

(2) $$\frac{1}{E_{r}}=\frac{1-v^{2}}{E}+\frac{1-{vi}^{2}}{E_{i}}$$

where E represents the sample modulus, ν denotes the Poisson ratio (0.35), E_r_ is the reduced modulus (acquired from the machine), Ei is the diamond indenter modulus (1141 GPa), and vi refers to the Poisson ratio of the indenter (0.07) [59].

### 2.10. Surface Wettability Analysis Using Water Contact Angle Measurement

The wettability characteristics of EP and EPGA overlays were assessed by measuring static water contact angles by the sessile drop method [60] at room temperature using twice-distilled water on the equipment (DMs-401, Kyowa Interface Science Co., Ltd., Niiza, Japan). A 2 µL liquid droplet formed at the end of the syringe and was carefully deposited onto the sample surface. A charge-coupled device (CCD) camera was utilized to capture an image of the static contact angle within 3 s after liquid deposition.

### 2.11. Surface Characterizations Using Optical Microscope, Energy Dispersive System (EDS)

An optical microscope (make: Olympus, model: BX53M) and a scanning electron microscope (make: ZEISS, model: EVO18) were utilized for detailed surface analysis. To enhance conductivity for SEM imaging, the coated samples were sputter-coated with a thin layer of gold. Additionally, the worn surfaces were further characterized using an energy-dispersive X-ray spectroscopy (EDS) system to analyse elemental composition. These analyses provide insights into the wear mechanisms of both EP and EPGA overlays.

### 2.12. Optimization of GA Concentration Using the TOPSIS MCDM Technique

Multi-response optimisation of the tribological performance was carried out using the Technique for Order Preference by Similarity to an Ideal Solution (TOPSIS), a well-established Multi-Criteria Decision-Making (MCDM) method [61]. In the present study, the Taguchi L16 orthogonal array was employed solely to generate the experimental matrix, whereas the optimisation of the tribological responses was performed entirely using TOPSIS. The optimisation considered two response parameters, namely the coefficient of friction (COF) and the specific wear rate (SPWR), both of which were required to be minimized. Equal weights (0.5 each) were assigned to COF and SPWR, as both responses were considered equally important in evaluating the tribological performance of the epoxy overlays. The experimental data were first normalized using the vector normalization method, followed by the construction of the weighted normalized decision matrix. Subsequently, the positive ideal solution (PIS) and negative ideal solution (NIS) were determined, and the Euclidean distances of each experimental alternative from the PIS and NIS were calculated. Finally, the closeness coefficient (CC) was determined using Equation (3), and the experimental combinations were ranked accordingly. The experimental condition with the highest CC value was considered the optimum parameter combination. A detailed description of the TOPSIS methodology, including the raw experimental data, decision matrix, vector normalization, weighted normalized matrix, positive and negative ideal solutions, separation distances, and closeness coefficient calculations for all 16 experimental trials, is provided in the Supplementary Information.

(3) $${CC}_{i\;}=\frac{S_{i}^{-}}{\;S_{i}^{+}+S_{i}^{-}}$$

where $S_{i}^{+}$ and $S_{i}^{-}$ denote the separation distances from the positive ideal and negative ideal solutions, respectively [62]. The methodology is provided in the Supplementary Information.

## 3. Results

### 3.1. Mechanical Properties of the Composite Overlays Using Nanoindentation

**Figure 5:**
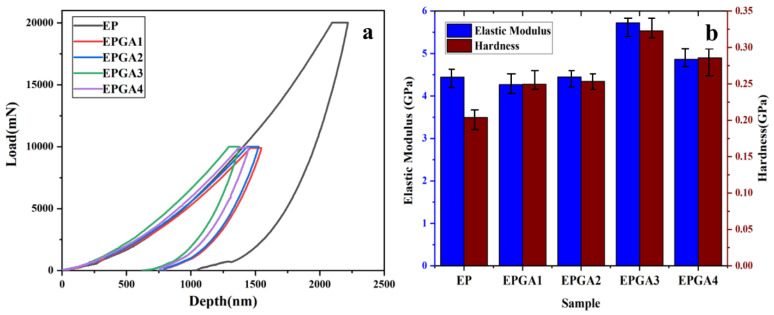
(**a**) Nanoindentation load vs. depth curves; (**b**) comparison of average elastic modulus vs. hardness for pure EP and different EPGA composites.

The nanoindentation results demonstrate that the incorporation of GA significantly influences the mechanical response of the epoxy overlays (Figure 5). The load-displacement curves (Figure 5a) illustrate the penetration behaviour of the indenter into each sample. Figure 5a shows the load vs. depth curves, which represent the penetration depth of the indenter under an applied load. The pure EP overlay exhibits the highest penetration depth (2150.02 ± 0.12 nm), indicating lower resistance to deformation and comparatively lower hardness. With the addition of GA, the curves shift towards lower indentation depths, confirming enhanced resistance to penetration. Among the composites, EPGA3 (1 wt.% GA) shows the lowest penetration depth (1320 ± 0.21 nm), indicating superior resistance to deformation. This improvement can be attributed to better dispersion of GA within the epoxy matrix, leading to effective stress transfer and improved load-bearing capability. In contrast, higher GA loading (EPGA4, 3 wt.%) shows a slight increase in penetration depth, which may be due to agglomeration of GA particles, resulting in localized stress concentrations. Furthermore, the unloading curves of GA-reinforced overlays exhibit steeper slopes compared to pure EP, indicating improved elastic recovery and stiffness. This suggests that GA incorporation enhances the viscoelastic behaviour of the overlay, allowing it to better recover after deformation.

Figure 5b exhibits the average elastic modulus and hardness values. Both properties increase with GA addition up to 1 wt.% (EPGA3), reaching maximum values of approximately 5.72 GPa (elastic modulus) and 0.32 GPa (hardness). The enhancement in hardness and elastic modulus observed for the GA-reinforced epoxy overlays can be attributed to the improved interfacial interaction between the GA particles and the epoxy matrix. The hydroxyl- and carboxyl-rich functional groups present in GA promote hydrogen-bonding interactions with the epoxy network, resulting in improved interfacial adhesion and more efficient stress transfer under localized loading. Consequently, the coating exhibits greater resistance to plastic deformation during indentation. A similar interface-controlled mechanical response has been reported by Lapčík et al. [63] for epoxy nanocomposites, who demonstrated that the mechanical performance of epoxy composites depends not only on the intrinsic properties of the reinforcing phase but also on homogeneous filler dispersion and strong filler–matrix interfacial bonding, which facilitate efficient load transfer and improve resistance to localized deformation. These findings are consistent with the present results, where the homogeneous dispersion of GA and the enhanced filler–matrix interfacial interactions contribute to the observed increase in hardness and elastic modulus. However, beyond the optimum GA concentration (1 wt.%), a slight reduction in both properties is observed for EPGA4, which may be attributed to particle agglomeration and the consequent reduction in effective interfacial bonding. Overall, the results indicate that an optimum GA content significantly enhances the mechanical integrity of the epoxy overlays by improving stiffness, hardness, and resistance to deformation, whereas excessive GA loading adversely affects the mechanical performance. Similar deterioration in mechanical properties at higher reinforcement contents has also been reported in previous studies [64,65].

Although the pull-off adhesion results indicate that all GA-reinforced overlays exhibit comparable adhesion strengths, the nanoindentation results clearly identify EPGA3 (1 wt.% GA) as the optimum composition owing to its highest hardness, elastic modulus, and lowest penetration depth. This suggests that the superior tribological performance of EPGA3 is governed primarily by its enhanced mechanical integrity and efficient stress transfer resulting from homogeneous GA dispersion, rather than by further improvements in adhesion strength. At higher GA loading (3 wt.%), particle agglomeration reduces reinforcement efficiency, leading to a slight deterioration in the mechanical properties despite maintaining comparable adhesion strength. Therefore, the optimum GA content of 1 wt.% is established based on the combined assessment of nanoindentation, tribological performance, and microstructural observations rather than adhesion strength alone.

### 3.2. Water Contact Angle of the EPGA Overlays

**Figure 6:**
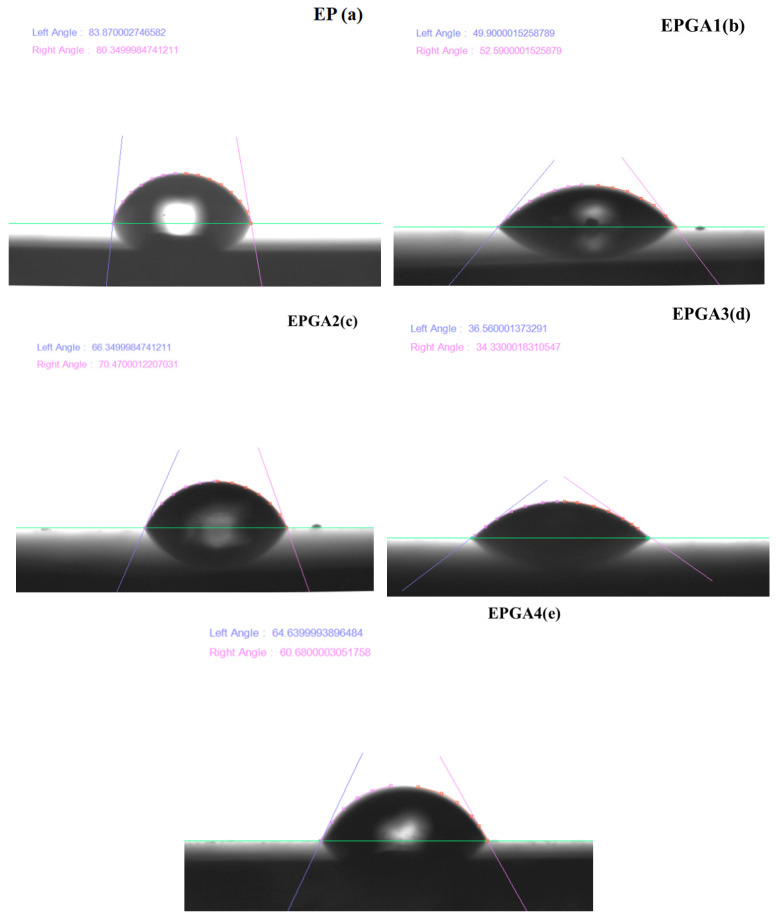
Water contact angle image: (**a**) EP, (**b**) EPGA1, (**c**) EPGA2, (**d**) EPGA3, and (**e**) EPGA4.

The water contact angle (WCA) measurements are presented in Figure 6. The pure epoxy (EP) overlay exhibited a relatively high contact angle of approximately 82.1°, reflecting the inherently low surface energy and hydrophobic nature of the cured resin [66].

With the incorporation of GA, a significant decrease in the water contact angle was observed, indicating a transition of the coating surface toward a more hydrophilic character. The water contact angle decreased from that of pure EP to 51.3° for EPGA1 and reached a minimum value of 35.5° for EPGA3 (1 wt.% GA). This enhanced wettability can be attributed to the presence of abundant polar functional groups in the GA structure, particularly hydroxyl (–OH) and carboxyl (–COOH) groups [67]. These polar groups increase the surface polarity of the coating and promote stronger hydrogen-bonding interactions with water molecules, thereby reducing the contact angle and improving the wetting behaviour of the composite overlay.

Interestingly, at the highest GA loading (EPGA4, 3 wt.%; Figure 6e), the contact angle increased slightly to 62.7°. This suggests that at higher concentrations, partial agglomeration of GA may occur, reducing the effective exposure of hydrophilic functional groups at the air-overlay interface. Alsunbul et al. [68] discovered that adding GA to glass ionomer cement reduced the water contact angle, suggesting enhanced hydrophilicity and improved surface interaction resulting from better dispersion and accessibility of polar groups at low concentrations. Sharkawy et al. [69] reported that, as the GA content increases, hydrophobic moieties can promote particle agglomeration, thereby affecting wettability and contact angle measurements. They reported that the contact angle of chitosan/GA nanoparticles increases with GA content due to the amphiphilic nature of GA. The biopolymeric structure of GA [70], which promotes polar interactions, is responsible for the reduction in surface free energy. Applications requiring coating adhesion, corrosion resistance, or bio-functionality can benefit from the enhanced surface wettability [71]. Although the tribological tests in the present study were conducted under dry sliding conditions, the reduced water contact angle should not be interpreted as a direct contributor to friction reduction or wear resistance. Instead, the increased wettability suggests improved interfacial interactions between GA and the epoxy matrix and may be associated with an increase in surface free energy [72]. This interpretation is supported by the FTIR results, which confirmed hydrogen-bond formation, and the pull-off adhesion measurements, which demonstrated improved interfacial bonding. Enhanced interfacial integrity promotes more efficient stress transfer, suppresses crack initiation and coating delamination, and thereby indirectly contributes to the improved tribological performance of the GA-reinforced epoxy overlays under dry sliding conditions.

### 3.3. Investigating the Tribological Properties of the Overlays

**Figure 7:**
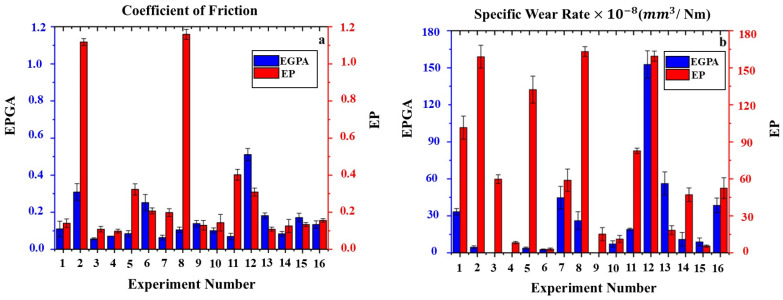
Comparison of EPGA and EP overlays: (**a**) COF; (**b**) SPWR.

**Figure 8:**
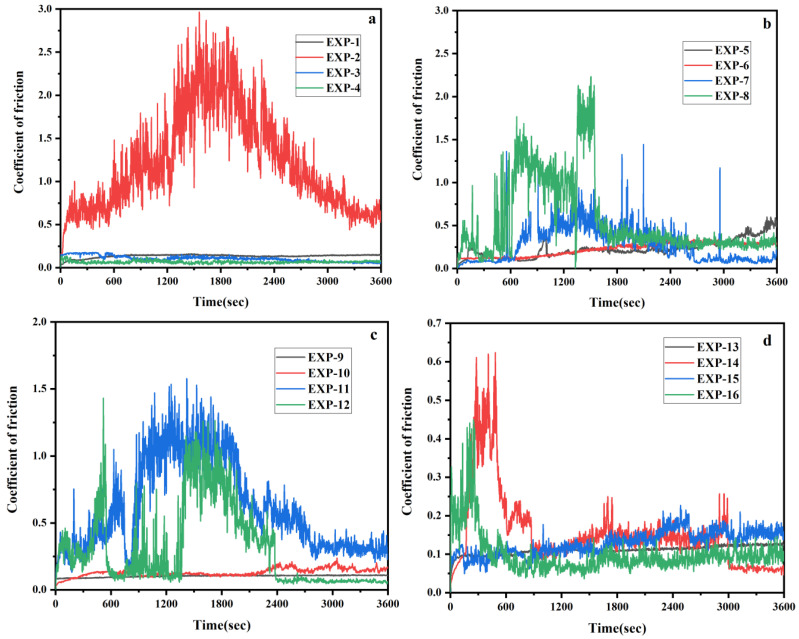
(**a**–**d**) COF vs. time graphs for different experimental conditions of EP overlays.

**Figure 9:**
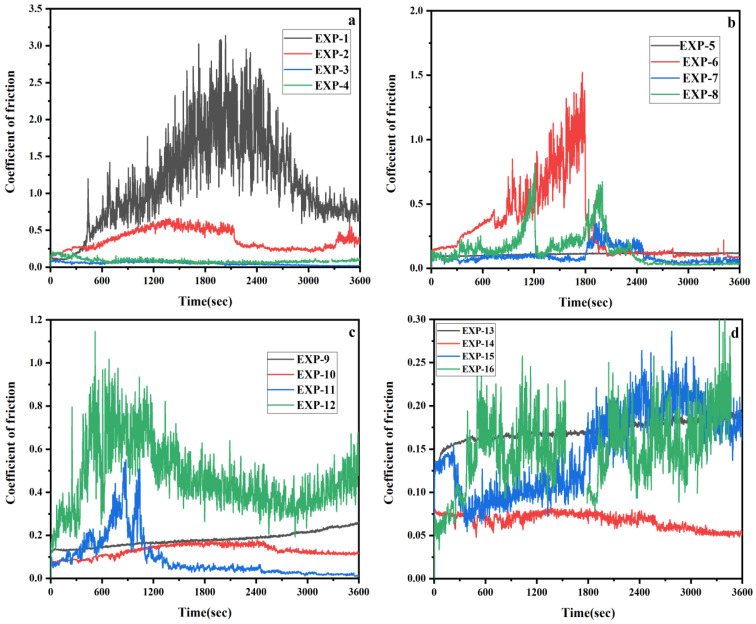
(**a**–**d**) COF vs. time graphs for different experimental conditions of EPGA overlays.

The tribological performance of the overlays was evaluated under the experimental conditions defined by the L16 Taguchi orthogonal array. The results revealed substantial variations in friction and wear behaviour, highlighting the significant influence of GA concentration, applied load, sliding frequency, and temperature on the tribological response of the coatings. Figure 7 presents a comparative analysis of the coefficient of friction (COF) and specific wear rate (SPWR) of the pure EP and GA-reinforced EP overlays, while Figure 8 and Figure 9 show the corresponding real-time COF–time evolution under different operating conditions. At lower applied loads (Experiments 1–4, 5 N), the EPGA overlays exhibited markedly improved tribological performance, with the 1 wt.% GA overlay (Exp 3) showing the lowest COF (0.0567 ± 0.0021) and negligible wear scar formation. This superior behaviour can be attributed to the formation of a stable tribo-film, improved load-sharing ability, and enhanced resistance to surface damage, even with increasing sliding frequency and temperature. In contrast, the pure EP overlays, particularly in Experiments 2 and 8, displayed a pronounced increase in both COF (1.11 ± 0.0034 and 1.15 ± 0.0023, respectively) and specific wear rate, indicating severe adhesive interaction, inadequate protection of the contact interface, and progressive peeling of the overlay under similar test conditions.

The EPGA overlays containing lower GA contents exhibited comparatively moderate tribological improvement (Figure 9). For example, Exp 1 (0.25 wt.% GA) showed a moderate COF (0.099 ± 0.0022) accompanied by noticeable wear, suggesting that the low GA concentration was insufficient to form a continuous and stable protective tribo-film. Increasing the GA content to 0.50 wt.% (Exp 2) resulted in a higher COF (0.3038 ± 0.0045) but a lower SPWR (Figure 7), indicating a slight improvement in wear resistance despite the persistence of abrasive wear scars. At the highest GA loading of 3 wt.% (Exp 4), the COF remained relatively low (0.07 ± 0.0027), with moderate friction and wear behaviour; however, the excessive GA content likely promoted particle agglomeration, thereby reducing the mechanical integrity and reinforcement efficiency of the overlay. These observations suggest that, under low applied load and elevated temperature (70 °C), GA can improve the lubricating behaviour of the epoxy overlay, but only when present at an optimum concentration that enables stable tribo-film formation without compromising coating integrity.

At intermediate loads (Experiments 5–8, 10 N), the EPGA overlays continued to exhibit improved tribological performance compared with the pure EP coating. In particular, Exp 5 containing 1 wt.% GA showed a low COF (0.084 ± 0.0047) with minimal wear, highlighting the beneficial effect of an optimum GA concentration. In contrast, both lower GA content (Exp 7, 0.25 wt.%) and higher GA content (Exp 6, 3 wt.%) resulted in a slight increase in COF and wear, which can be attributed to either insufficient tribo-film formation at low GA loading or particle agglomeration and poor dispersion at excessive GA loading. Under the corresponding test conditions, the pure EP overlays consistently exhibited higher wear rates and unstable friction behaviour, primarily due to progressive surface deterioration. The SPWR data further confirm that GA plays a critical role in reducing material loss. In particular, the pure EP overlays exhibited elevated wear rates and higher COF values in Experiments 2, 8, 11, and 12 (Figure 7), indicating their susceptibility to severe material removal under reciprocating sliding. Similar behaviour has also been reported by Baig and Samad [73], where insufficient reinforcement in epoxy coatings led to stress concentration, crack formation, and accelerated wear.

At higher applied loads (Experiments 9–12, 15 N), the beneficial effect of GA became even more evident. The EPGA overlay containing 1 wt.% GA (Exp 10) consistently exhibited excellent tribological performance, maintaining a low COF (0.06887 ± 0.0033) and low wear rate despite the increased severity of the operating conditions. However, at lower GA loading (Exp 12, 0.25 wt.%), a comparatively high wear rate was observed, which may be attributed to inadequate filler dispersion and weak interfacial bonding, thereby compromising the structural integrity of the overlay. Similarly, at higher GA loading (Exp-9, 3 wt.%), increased friction and pronounced wear were observed, likely due to particle agglomeration, local stress concentration, and the associated plastic deformation and cracking. These observations clearly highlight the importance of optimising the GA concentration for effective reinforcement. Under the same conditions, the pure EP overlays without GA exhibited severe delamination and unstable frictional behaviour.

At the highest applied loads (Experiments 13–16, 20 N), the optimised EPGA overlay containing 1 wt.% GA (Exp 16) continued to maintain relatively low friction and wear, demonstrating improved resistance to both mechanical and thermal stresses, although minor crack formation was still observed. In contrast, Exp 13 containing 0.5 wt.% GA exhibited the poorest tribological performance among the EPGA overlays, with a sharp increase in both friction and wear rate, indicating inadequate tribo-film formation and poor load distribution under severe operating conditions. Likewise, the pure EP overlays in Experiments 13–15 showed elevated friction, excessive wear, and extensive surface failure. The absence of GA in the pure EP coating resulted in dry sliding conditions with poor load-bearing capacity, thereby causing severe fluctuations in COF and SPWR.

The superior tribological behaviour of the GA-reinforced overlays can be attributed to the combined effects of improved mechanical integrity, stable tribo-film formation, and enhanced thermal resistance. The TGA results demonstrated that GA incorporation increased both the onset degradation temperature and the char yield of the epoxy system, indicating improved thermal stability. This enhanced thermal resistance helps to preserve the integrity of the tribo-film under frictional heating, thereby reducing direct asperity contact, minimizing material removal, and stabilising the friction response during sliding [74]. In contrast, the pure EP overlays exhibited larger fluctuations in COF and severe peeling, which can be correlated with their lower thermal stability and lower char residue observed in the TGA analysis. Overall, the comparative results clearly demonstrate that GA-reinforced epoxy overlays provide superior tribological performance compared with pure epoxy coatings by reducing friction, minimizing wear, stabilising the sliding response, and improving structural integrity. These benefits arise from the film-forming nature of GA, its load-bearing contribution, and its ability to promote the formation of stable protective tribo-layers, thereby establishing GA as a promising bio-based additive for enhancing epoxy overlay systems.

To establish a quantitative relationship between the nanomechanical behaviour and tribological performance of the overlays, the nanoindentation results were correlated with the measured coefficient of friction (COF) and specific wear rate (SPWR). A clear trend was observed as the overlays with higher hardness and elastic modulus and lower indentation depth exhibited superior tribological performance. In particular, EPGA3 (1 wt.% GA), which showed the highest hardness (~0.32 GPa) and elastic modulus (~5.72 GPa) together with the lowest penetration depth (~1320 nm), also exhibited the lowest COF (0.0567 ± 0.0021) and negligible wear under the optimum sliding condition. In contrast, pure EP, which showed the highest penetration depth (~2150 nm) and the lowest hardness and modulus, exhibited the poorest tribological behaviour, with severe adhesive wear, delamination, and much higher friction. This correlation indicates that improved nanomechanical properties directly contribute to better tribological performance by increasing the load-bearing capacity of the overlay, suppressing localized plastic deformation, and reducing crack initiation and coating failure during sliding. The reduction in penetration depth for EPGA3 further confirms its greater resistance to subsurface deformation, which helps maintain the integrity of the tribo-contact and promotes the formation of a stable protective transfer layer. Although EPGA4 also exhibited relatively high hardness and modulus, its tribological performance was slightly inferior to EPGA3, which can be attributed to GA agglomeration at higher filler loading, resulting in localized stress concentration and reduced reinforcement efficiency. Therefore, the optimum tribological performance of EPGA3 can be quantitatively associated with its optimum nanomechanical response, demonstrating that hardness, elastic modulus, and deformation resistance are key governing parameters for the wear resistance of the GA-reinforced epoxy overlays.

### 3.4. Statistical Analysis of the Tribo Results Using Analysis of Variance

The experimental data were analysed using ANOVA, or analysis of variance, a statistical method for comparing means among various groups to determine the presence of any significant differences. The analysis was employed to evaluate the influence of GA addition on the COF and SPWR of the EP composites. The ANOVA results are presented in Table 6. The results demonstrate that the low *p*-values, all significantly (<0.05) [75] indicate a significant effect of material type on both the COF and SPWR properties. The notably small *p*-values for COF in both EP (*p* = 8.29 × 10^−11^) and EPGA (*p* = 6.27 × 10^−10^) indicate significant statistical effects. Similarly, notable *p*-values are observed for SPWR (*p* = 0.0177 for EP; *p* = 2.60 × 10^−7^ for EPGA), indicating that the inclusion of GA significantly impacts wear performance. The mean square (MS) findings indicated that the addition of GA decreased the variation in COF by around 87.4% and in SPWR by 50.5% relative to pure EP overlays. This decrease indicates that GA reinforcement enhanced the wear resistance and frictional stability of the overlays while also reducing variations under diverse tribological circumstances. The high F-values and low *p*-values show that differences in means are not just random. Adding GA changes, the characteristics of the matrix, making it less hard, tougher to break, and better at tribological performance like COF and SPWR. Alsadon et al. [76] studies indicate that plant gums and fillers, such as GA, can markedly affect the multi-response performance indicators of EP composites, frequently resulting in alterations in both friction and wear characteristics. These alterations are due to changes in the composite microstructure’s interface and load-transfer processes.

### 3.5. Optimizing the GA Content to Achieve the Least COF and SPWR

The multi-criteria decision-making study employing the TOPSIS approach confirmed that the formulation with 1% GA in Experiment 3, which is (5 N load, 2 Hz frequency, 60 °C, 1 wt.% GA), consistently achieved the top ranking based on the combined coefficients of friction and wear resistance performance. Additionally, indentation analyses validated that this optimised composition had superior mechanical characteristics, including increased hardness and modulus, hence enhancing load-bearing capacity and resistance to surface damage. The findings confirm that an optimal GA concentration, specifically 1 wt.%, provided superior mechanical and tribological conditions, enhancing overlay performance, making it an appropriate choice for practical applications. The details of the optimisation are provided in the Supplementary Information.

## 4. Exploring the Wear Mechanisms and Failure of the Overlays

The wear behaviour observed in the present study involved a combination of adhesive wear, abrasive wear, and third-body interactions, all of which were strongly influenced by the operating conditions and overlay composition. The pure EP overlays predominantly exhibited adhesive wear, characterized by strong interfacial interaction with the counterface, material transfer, and progressive surface degradation. In contrast, the wear mechanism of the EPGA overlays was more complex and was governed by multiple factors, including GA concentration, applied load, sliding frequency, temperature, and the mechanical properties of the overlay such as hardness and elastic modulus. Temperature played a particularly important role in determining the wear response, as increasing temperature generally reduced the hardness and load-bearing capability of the epoxy matrix, thereby promoting thermal softening and enhanced material removal during sliding [77]. Unlike metals, where plastic deformation is governed by dislocation motion, the tribological behaviour of epoxy coatings at elevated temperatures is controlled by viscoelastic deformation, increased molecular chain mobility, and frictional softening of the polymer matrix. These mechanisms reduce the load-bearing capacity of the coating and promote plastic deformation and wear under sliding conditions [78].

**Figure 10:**
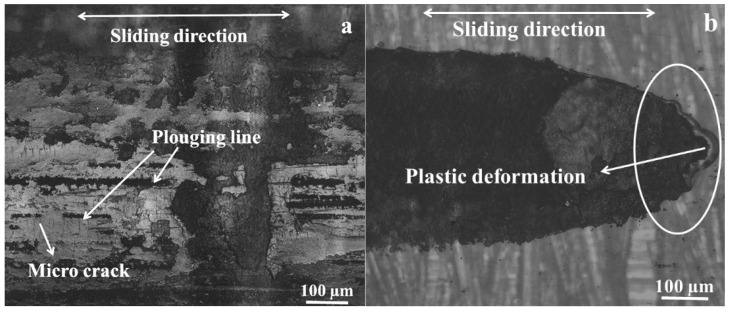

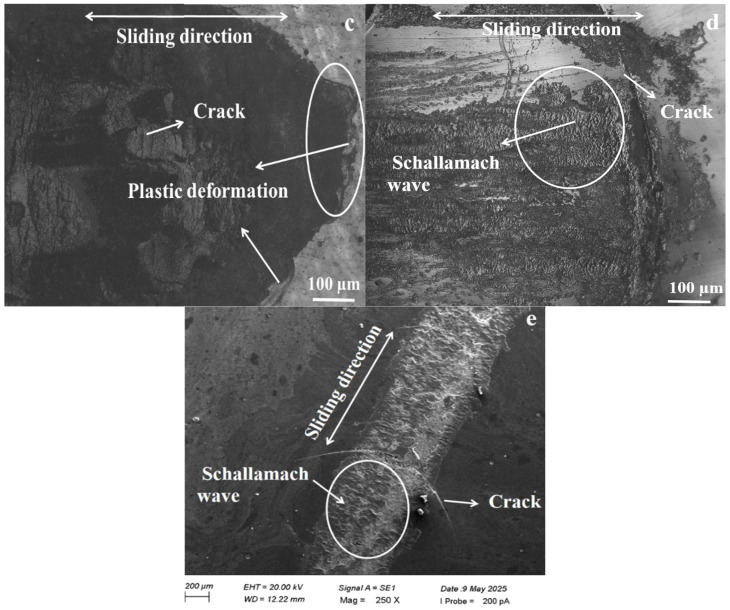
Optical images of EPGA overlay showed ploughing line at (**a**) 5 N, 1 Hz, 40 °C, GA (0.25%); (**b**) EP overlay at 20 N, 2.5 Hz, 40 °C; (**c**) EPGA overlay at 15 N, 2.5 Hz, 50 °C, showing plastic deformation; (**d**) EPGA overlay showing a Schallamach wave pattern at 20 N, 2 Hz, and 50 °C; (**e**) SEM image of the Schallamach wave pattern of the EPGA overlay at 20 N, 2 Hz, and 50 °C.

At low GA loading, such as Exp 1 (0.25 wt.% GA), the EPGA overlays exhibited ploughing marks and microcracks (Figure 10a), indicating that the reinforcement was insufficient to form a stable protective tribo-layer. For the pure EP overlays, plastic deformation was relatively limited under mild operating conditions (5 N, 1 Hz, 40 °C), whereas under more severe conditions involving higher load and frequency, significant plastic deformation and surface damage were observed (Figure 10b). The combined effect of increased load and sliding speed generated greater frictional heat, which softened the epoxy matrix, reduced its structural integrity, and promoted localized plastic deformation and material removal. In comparison, the EPGA overlays exhibited a more stable deformation response due to the presence of GA. Under conditions such as 15 N, 2.5 Hz, 0.25 wt.% GA, and 50 °C, the EPGA overlay underwent plastic deformation and material redistribution (Figure 10c), but the GA-modified viscoelastic matrix accommodated the imposed stresses through localized ductile flow rather than catastrophic brittle failure. The GA-rich regions helped to arrest and stabilise microcracks, thereby reducing the likelihood of sudden peeling and large-scale delamination. As a result, the worn EPGA surfaces displayed fewer deep grooves and smoother Schallamach wave-like features (Figure 10d,e), indicating improved surface compliance and controlled deformation during sliding. Overall, the incorporation of GA enhanced the ability of the epoxy matrix to absorb and dissipate sliding-induced stresses, thereby improving wear resistance and maintaining structural integrity under tribological loading [79].

**Figure 11:**
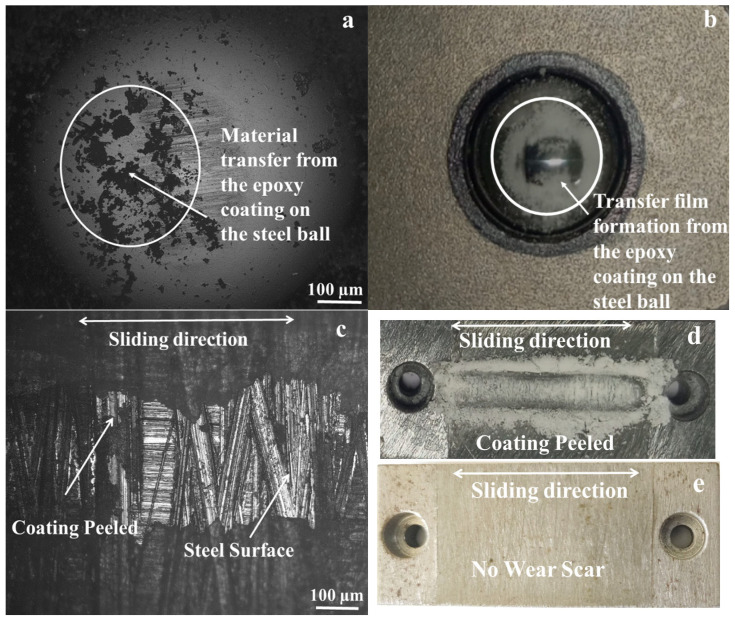
(**a**) Optical microscopic images of transfer film formation of the steel ball. (**b**) Photographic image of the counterbody indicating the transfer film formation of the steel ball. (**c**) Optical microscopic image of removal of the overlay on the steel surface. (**d**) Photographic image of overlay removal from the steel bar (**e**) Photographic image of the EPGA-overlayed bar after the tribological test.

The lack of a tribo-film in EP samples resulted in direct contact between asperities, which heightened both frictional resistance and surface roughness. This behaviour is generally linked to adhesive wear and the effects of interfacial shearing. The formation of a transfer film affects the wear behaviour [80] between the mating pair, as this adhesive wear results in regions of high COF. When the hard EN31 steel ball slides over the softer EP overlay, the difference in hardness causes adhesive wear, resulting in epoxy film transfer on the steel surface (Figure 11a,b). Most of the polymer overlays adhere to the countersurface by Van der Waals forces. In most wear situations, this form of adhesion is not strong enough for lumps of material to be torn out on rupture of this contact [81,82]. In particular, EP overlays exhibited elevated COF values in Exp 2 and 8, indicating inadequate lubrication and significant adhesive forces between the overlay and the counterface, leading to peeling of the overlay (Figure 11c,d). The presence of GA in EPGA overlays prevents adhesive wear by acting as a natural lubricant [83]. This lubrication effect reduces the friction between sliding surfaces, resulting in consistently lower COF compared to the pure epoxy overlay. As a result, the EPGA overlays experience less surface resistance during sliding (even no visible wear scars, Figure 11e), enhancing their tribological performance.

Additionally, pure EP exhibited significant variations in COF throughout the tests, indicating inconsistent tribological performance that may be due to inadequate load distribution, surface fatigue, and thermal degradation [84]. On the other hand, EPGA overlays, especially those with ideal GA content, exhibited notably smoother frictional behaviour. The reduction in COF can be attributed to the film-forming and lubricating properties of GA, which facilitate the formation of a tribo-protective layer. This layer diminishes the contact between metal and overlay, thereby minimizing interfacial shear stresses. The presence of GA contributes to a more uniform contact surface and effective energy dissipation, thereby enhancing lubrication under dynamic conditions. In a series of experiments, GA-reinforced overlays exhibited stable COF values, indicating enhanced structural integrity and reliable performance. The role of GA in these overlays is that of a physically bonded additive, indicating that the GA particles are not chemically cross-linked within the EP matrix but are rather physically dispersed.

**Figure 12:**
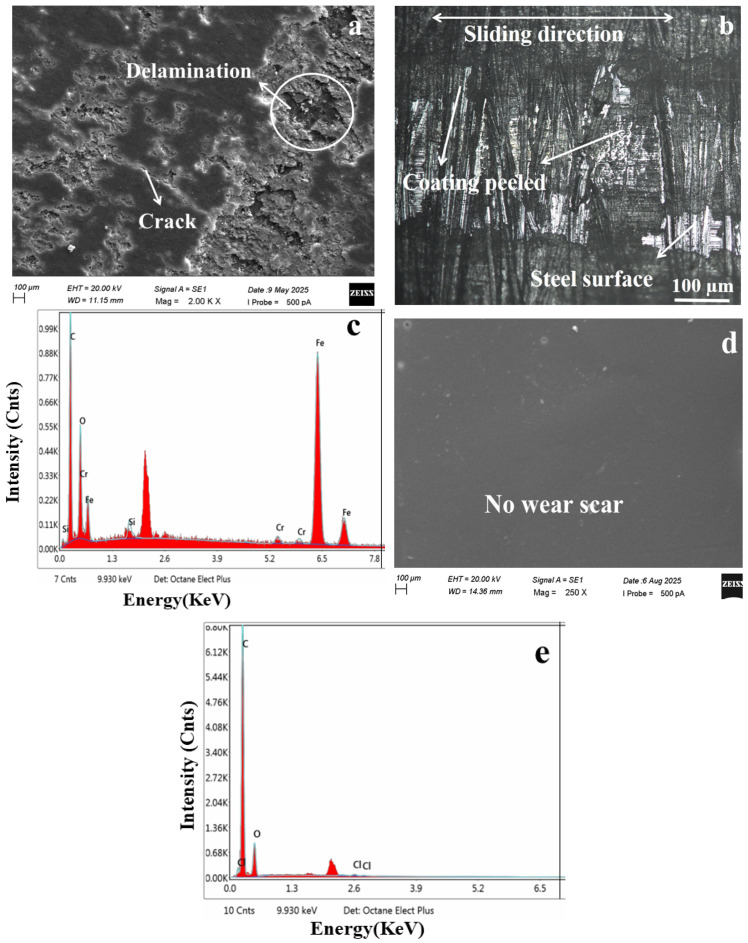
SEM image of EP overlay at (**a**) 5 N, 1.5 Hz, 50 °C showing delamination and cracking; (**b**) optical image of EP overlay at 15 N, 1 Hz, 60 °C, showing peeling of the overlay; (**c**) EDS of EP overlay at 15 N, 1 Hz, 60 °C. SEM image indicating the EPGA-overlayed surface at (**d**) 5-N load, 2 Hz frequency, 1%GA, 60 °C. (**e**) EDS of EPGA at 5 N load, 2 Hz frequency, 1%GA, 60 °C.

The EP overlays have high COF due to their inherent brittleness and limited self-lubricating characteristics, which result in more direct contact and interactions between surface asperities during sliding. This increased asperity contact promotes abrasive wear. Abrasive wear occurs when hard bodies or asperities slide against a softer surface, causing material removal through mechanical action. The process can generate wear debris and surface damage such as grooves, cracks, and material detachment [85,86]. In this work, the steel counter surface slid against the epoxy overlay, resulting in grooves, cracks, and even delamination of the overlay. This is mainly due to the increased friction between the hard surface and soft surface. The EP overlay tested under 5 N, 1.5 Hz, and 50 °C conditions (Exp. 2) exhibited a significant increase in both the coefficient of friction (COF: 1.11 ± 0.0034) and specific wear rate (SPWR), indicating severe surface degradation. The elevated friction and wear promoted delamination of the overlay, as observed in Figure 12a. Similarly, the EP overlay subjected to 15 N, 2 Hz, and 60 °C conditions (Exp. 8) showed a substantial increase in COF (1.15 ± 0.0023) and SPWR, suggesting inadequate lubrication and intensified adhesive interactions at the contact interface. These conditions accelerated overlay peeling and material removal, as illustrated in Figure 12b.

The EDS spectrum of the EP overlay (Figure 12c) exhibited prominent peaks corresponding to iron (Fe), along with minor peaks for chromium (Cr) and manganese (Mn), which originate from the underlying EN8 steel substrate. The appearance of strong metallic signals indicates that the EP overlay experienced significant wear and peeling of the overlay, leading to complete exposure of the steel substrate. The lower carbon and oxygen content in this spectrum further confirms the peeling of overlay material at the analysed location.

In contrast, the addition of GA influences the tribological response of EP overlays by creating a natural polymeric matrix that affects interfacial interactions. The polysaccharide composition of GA improves load distribution and creates a smoother contact surface, lowering friction and increasing wear resistance [87]. The experimental data show that GA-modified overlays had a lower COF value and no wear scar (Figure 12d) than EP, indicating improved lubrication due to the creation of a transfer film or a modified surface layer that reduces direct contact between sliding surfaces. Furthermore, the concentration of GA has a considerable impact on the wear rate of the overlays, as optimal load-bearing capability is reached with modest reinforcement levels. Excess GA content can cause agglomeration, decreasing the homogeneity of the overlay and affecting its mechanical stability, which lowers wear resistance [88]. The EDS spectrum of the EPGA composite overlay (Figure 11e) shows dominant peaks corresponding to carbon (C) and oxygen (O) [89], which are primarily attributed to the organic nature of the EP resin and the GA additive. The relatively high carbon content indicates the presence of intact polymeric matrix material on the wear surface. The absence of significant peaks for metallic elements such as iron (Fe) suggests that the overlay layer largely remained intact during the wear process, thereby preventing direct exposure of the steel substrate. This indicates that the incorporation of GA improved the overlay’s wear resistance and protective ability under sliding conditions. The reduced wear rate for optimal EPGA formulations can be due to a combination of mechanical interlocking, improved adhesion, and surface film generation, all of which reduce material loss. Furthermore, the presence of GA enhanced the thermal and oxidative stability of the overlays, which improves their tribological performance at high temperatures [90]. The variation in COF and SPWR across different experimental conditions demonstrates the role of GA as a functional additive that modifies the deformation and failure mechanisms of EP overlays, resulting in improved tribological behaviour and potential applicability in wear-intensive environments. The lubrication and tribo-film formation, resulting from GA’s hydrophilic nature and film-forming ability, contribute to friction reduction by forming a lubricating layer [91]. This layer acts as a buffer, reducing the shear stress at the contact interface. The self-lubricating properties of GA help in distributing the applied load more effectively, preventing excessive wear.

## 5. Conclusions

In the present study, gum arabic (GA), a natural bio-based polysaccharide, was successfully incorporated into epoxy (EP) overlays deposited on EN8 steel substrates to improve their mechanical, thermal, adhesive, and tribological performance. The results demonstrate that GA significantly influences the physicochemical and tribological behaviour of epoxy overlays, and that the extent of improvement strongly depends on the GA loading.

FTIR analysis confirmed the retention of the characteristic functional groups of both EP and GA, while the broadening of the O–H band and the reduction in the epoxide peak intensity indicated intermolecular interactions and hydrogen bonding between the GA particles and the epoxy network. These interactions improved the compatibility between the two phases and contributed to the enhanced mechanical and tribological behaviour of the overlays. Water contact angle measurements further revealed a marked reduction in contact angle from 82.1° for pure EP to 35.5° for EPGA3, indicating increased surface polarity and wettability due to the hydroxyl- and carboxyl-rich nature of GA. Although this increased wettability is not directly responsible for improved dry sliding performance, it reflects improved interfacial interactions and stronger matrix–filler compatibility, which indirectly contribute to better coating integrity and wear resistance.

Thermal analysis demonstrated that GA incorporation significantly improved the thermal stability and thermal transition behaviour of the epoxy overlays. Thermogravimetric analysis showed that the onset degradation temperature increased progressively from 320 °C for pure EP to 342 °C for EPGA4, while the final char residue increased from approximately 6% in pure EP to nearly 20% in EPGA4, confirming the beneficial carbonaceous barrier effect of GA. Differential scanning calorimetry further showed that GA strongly influenced the glass transition behaviour of the epoxy matrix. The pure EP coating exhibited a glass transition temperature of approximately 115 °C, whereas EPGA3 showed the highest Tg of approximately 118 °C, indicating the most effective restriction of polymer-chain mobility due to improved interfacial interactions and homogeneous dispersion of GA. However, a further increase in GA loading to 3 wt.% reduced the Tg to approximately 110 °C, suggesting that excessive GA promotes agglomeration and reduces reinforcement efficiency.

Nanoindentation results confirmed that GA significantly enhanced the mechanical integrity of the overlays. The EPGA3 overlay exhibited the lowest penetration depth and the highest elastic modulus and hardness, indicating improved resistance to localized deformation and superior load-bearing capability. The enhancement in mechanical performance is attributed to the improved filler–matrix interfacial bonding and efficient stress transfer resulting from the hydrogen-bonding interactions between GA and the epoxy network. Pull-off adhesion testing further showed that the incorporation of GA nearly doubled the adhesion strength compared with pure EP, increasing from 10.34 ± 0.14 MPa for EP to approximately 20 MPa for the GA-reinforced overlays. Although all GA-modified overlays exhibited comparable adhesion strengths, the combined nanoindentation, tribological, and microstructural results clearly identified EPGA3 as the optimum composition.

Tribological evaluation under reciprocating sliding conditions revealed that the incorporation of GA markedly improved the friction and wear performance of the epoxy overlays. The EPGA3 overlay containing 1 wt.% GA exhibited the best overall tribological behaviour and was identified as the optimum formulation. Under the condition of 5 N load, 2 Hz frequency, and 60 °C, EPGA3 exhibited the lowest coefficient of friction of 0.0567 ± 0.0021 and negligible specific wear, whereas the pure EP overlay exhibited severe adhesive wear, peeling, delamination, and a maximum specific wear rate of 163 × 10^−8^ mm^3^/Nm under comparable severe conditions. The superior tribological performance of EPGA3 is attributed to the synergistic improvement in hardness, elastic modulus, interfacial integrity, thermal stability, and tribo-layer stability, which together enhanced load distribution, delayed crack initiation, reduced coating delamination, and improved resistance to material removal during sliding. In contrast, excessive GA loading (3 wt.%) led to a slight deterioration in performance, which is attributed to particle agglomeration and reduced reinforcement efficiency.

Overall, the present study establishes GA as a promising sustainable bio-based reinforcement for epoxy overlays. An optimum GA loading of 1 wt.% provides the best balance of thermal stability, glass transition behaviour, mechanical strength, adhesion, and tribological performance. The developed GA-reinforced epoxy overlay should therefore be regarded as a promising laboratory-scale protective tribological coating formulation for moderate dry sliding applications. However, further investigations involving long-term durability, humidity and corrosion exposure, scratch resistance, impact loading, fatigue, ageing behaviour, and application-specific service-life testing will be required before practical industrial implementation.

### Future Prospects

Future work should focus on detailed characterisation of the interfacial tribo-layer using advanced techniques to better understand the chemical and structural mechanisms governing friction reduction. In addition, long-term durability studies, including extended sliding cycles, fatigue loading, corrosion resistance, humidity exposure, scratch resistance, ageing behaviour, and service-life evaluation, are necessary to assess the stability and reliability of the developed overlays under realistic operating conditions. Furthermore, optimisation of overlay thickness will be carried out to balance tribological performance with practical industrial constraints. Future studies will also explore hybrid reinforcement strategies by combining GA with other nano- or micro-fillers to further enhance the mechanical and tribological performance of the epoxy overlays. Finally, validation of the developed overlays in application-specific environments, such as bearing systems, bushings, mining components, and other industrial tribological systems, will be essential to bridge the gap between laboratory-scale investigations and practical engineering applications.

## Figures and Tables

**Table 1:** Composition of EN8 and EN31.

Tribo-Pairs	Elements (wt.%)					
	C	Mn	Si	S	P	Cr
Steel bar, EN8	0.440%	0.569%	0.176%	0.027%	0.080%	---
Countersurface ball, EN31	0.928%	0.323%	0.186%	0.006%	0.019%	1.494

**Table 2:** Experiment design using an L16 array.

Exp	Load (N)	Frequency (Hz)	Temperature (°C)	GA (wt.%)
1	5	1	40	0.25
2	5	1.5	50	0.50
3	5	2	60	1
4	5	2.5	70	3
5	10	1	50	1
6	10	1.5	40	3
7	10	2	70	0.25
8	10	2.5	60	0.50
9	15	1	60	3
10	15	1.5	70	1
11	15	2	40	0.50
12	15	2.5	50	0.25
13	20	1	70	0.50
14	20	1.5	60	0.25
15	20	2	50	3
16	20	2.5	40	1

**Table 3:** Onset degradation temperatures (°C) of EP and EPGA overlay samples.

Sample	Onset Degradation Temperature (°C)
GA Powder	97
EP	320
EPGA1	325
EPGA2	330
EPGA3	336
EPGA4	342

**Table 4:** Estimated glass transition temperatures (Tg, °C) for the coated sample of EP and EPGA coatings.

Sample	Estimated Tg (°C)
GA Powder	~87
EP	~115
EPGA1	~100
EPGA2	~105
EPGA3	~118
EPGA4	~110

**Table 5:** Adhesion strength values of pure EP and GA-EP composite overlays.

Sample	Dolly1 (MPa)	Dolly2 (MPa)	Dolly3 (MPa)	Average Adhesion Strength (MPa)
EP	10.20	10.48	10.33	10.34 ± 0.14
EPGA1	20.54	20.98	20.56	20.69 ± 0.19
EPGA2	20.19	19.01	20.94	20.04 ± 0.80
EPGA3	20.86	19.15	20.98	20.33 ± 0.86
EPGA4	20.34	19.58	20.96	20.29 ± 0.69

**Table 6:** Analysis of variance (ANOVA) results for the COF and SPWR of pure EP (EP) and pure EP + GA (EPGA).

Sample	ANOVA				
	DF	SS	MS	F	*p*
COF for (EP) Pure EP	15	3.386	0.225	53.626	8.290 × 10^−11^
SPWR for (EP) Pure EP	15	9.080 × 10^−12^	6.053 × 10^−13^	3.016	0.017
COF for (EPGA) Pure EP + GA	15	0.426	0.028	41.223	6.273 × 10^−10^
SPWR for (EPGA) Pure EP + GA	15	4.490 × 10^−12^	2.993 × 10^−13^	18.406	2.601 × 10^−7^

DF = degrees of freedom, SS = sum of squares, MS = mean square, F = F-value, *p* = *p*-value.

## Data Availability

The original contributions presented in this study are included in the article/Supplementary Material. Further inquiries can be directed to the corresponding authors.

## Supplementary Materials

The following supporting information can be downloaded at: https://www.mdpi.com/article/10.3390/polym18141695/s1, Table S1: DOE-based experimental responses; Table S2: Normalized matrix; Table S3: Normalizing weight matrix; Table S4: Positive and Negative ideal solution; Table S5: Separation matrix; Table S6: Closeness coefficients value with rank order. Refs. [92,93,94,95] cited in Supplementary Materials.
