# Supplementary material for: Bio-Based Gum Arabic-Reinforced Epoxy Overlay System: Mechanical, Thermal, and Tribological Performance with Wear Mechanism Analysis
Source: Polymers (Basel). 2026 Jul 9;18(14):1695. doi: 10.3390/polym18141695 (PMC13417316; doi:10.3390/polym18141695)
Supplement: Supplementary file 1 [file polymers-18-01695-s001.zip › polymers-4407498-supplementary.pdf]

TOPSIS (MCDM)

S1.1. Technique for Order Preference by Similarity to an Ideal Solution (TOPSIS):

The Technique for Order Preference by Similarity to an Ideal Solution (TOPSIS) was initially introduced by Hwang and Yoon in 1981 and then enhanced by Yoon and Kin in 1987, as well as by Hwang, Lai, and Liu in 1993. This multi-criteria decision-making methodology is based on the principles of the positive ideal solution (PIS) and the negative ideal solution (NIS). The PIS denotes the theoretical best outcome, whilst the NIS indicates the theoretical worst outcome, with all experimental outcomes lying between these limits. The fundamental concept of TOPSIS is to determine the alternative that is nearest to the positive ideal solution (PIS) and farthest from the negative ideal solution (NIS) by computing a proximity coefficient. The method can be mathematically articulated as follows [1–3].

Step-I: Normalization the matrix using the following equation:

$$T_{ij} = \frac{X_{ij}}{\sqrt{\sum_{i=1}^{m} x_{ij}^{2}}} \quad \text{(S1)}$$

Xij denotes the actual value of the ith element of the jth variable.

Step II: The weight of each response is distributed equally between the coefficient of friction (COF) and the specific wear rate (SWR).

Step III: The normalization matrix is multiplied by the corresponding weights to obtain the weighted normalized decision matrix. The following formula establishes the weighted normalized decision matrix:

$$Vij = Wi \times Tij \quad \text{(S2)}$$

Where i=1, 2,…..m

j=1, 2,…..n ,

w j represents the weight of the jth attribute

Step IV: The positive ideal solution (V+) signifies the optimal value, whereas the negative ideal solution (V-) indicates the least desirable value for each attribute obtained from the weighted choice matrix.

$V^+ = (V_1 + V_2 + V_3 + \ldots\ldots. + V_n^{\ +})$ Maximum Values (S3)

$V^- = (V_1 - V_2 - V_3 - \ldots\ldots. - V_n^{\ -})$ Minimum Values (S4)

Step V: The separation distance of each solution, specifically the positive ideal solution (S+) and the negative ideal solution (S-), is calculated using the following equation.

$$Si^+ = \sqrt{\sum_{j=1}^{n}(Vij - Vj^+)^2} \quad \text{(S5)}$$

$$Si^- = \sqrt{\sum_{j=1}^{n}(Vij - Vj^-)^2} \quad \text{(S6)}$$

Step-VI: The closeness coefficient (CCO) is obtained using the following equation:

$$CC_O = \frac{Si-}{Si+ \ + \ Si-} \quad \text{(S7)}$$

Step-VI: The CCo result is ranked.

The CCo values are ranked in ascending order according to the closeness coefficient.

Table S1: DOE-based experimental responses:

Sl.no	Load	RPM	Temperature	GA(%)	COF (X)	SWR(Y)
E1	5	1	40	0.25%	0.09167	3.33362934774247E-07
E2	5	1.5	50	0.50%	0.3082	4.55321743436542E-08
E3	5	2	60	1.00%	0.0567	0
E4	5	2.5	70	3.00%	0.0614	0
E5	10	1	50	1%	0.084	3.74312E-08
E6	10	1.5	40	3.00%	0.2018	2.64869E-08
E7	10	2	70	0.25%	0.0695	4.47347E-07
E8	10	2.5	60	0.50%	0.1387	2.60773E-07
E9	15	1	60	3.00%	0.1512	1.19699E-09

E10	15	1.5	70	1%	0.1322	7.20227E-08
E11	15	2	40	0.50%	0.0688	1.90177E-08
E12	15	2.5	50	0.25%	0.5109	1.52653E-06
E13	20	1	70	0.50%	0.1814	5.6251E-07
E14	20	1.5	60	0.25%	0.0833	1.09983E-07
E15	20	2	50	3.00%	0.1712	8.95629E-08
E16	20	2.5	40	1%	0.1336	3.84815E-07

S1.2 Results and discussions:

This study applies the TOPSIS method to enhance multi-machining features. The objective of this approach is to obtain a single numerical value by calculating the two responses: the coefficient of friction (COF) and the specific wear rate (SWR). Based on the findings presented in Table 1, the results are initially transformed into a decision matrix format. Equation 1 in Table 2 is employed to determine the normalized matrix from Table 1. COF and SWR are considered as equally valuable attributes.

According to the decision of the discussion facilitator, the positive ideal solution (V+) for COF is represented by the lowest COF value, while the negative ideal solution (V-) is represented by the highest COF number. For SWR, the positive ideal solution (V+) is represented by the lowest number, while the negative ideal solution (V-) is represented by the highest value. The values derived from equations 3 and 4 are presented in Table 4. Table 5 presents the values. Equation 7, presented in Table 6, is utilized to get the closeness coefficients (CCO). The CCo are ranked according on their performance. The optimal parameter combination is characterized by a higher CCo value.

Table S2. Normalized matrix:

Sl.no	COF (X)	SWR(Y)
E1	0.0771633	7.51359E-05
E2	0.259483726	1.02624E-05
E3	0.047755331	0
E4	0.051683502	0

E5	0.070707071	8.43654E-06
E6	0.16986532	5.96983E-06
E7	0.058501684	0.000100827
E8	0.116806958	5.87751E-05
E9	0.127314815	2.69786E-07
E10	0.111335578	1.6233E-05
E11	0.05797138	4.28635E-06
E12	0.430050505	0.000344062
E13	0.152693603	0.000126783
E14	0.070159933	2.47888E-05
E15	0.144111953	2.01864E-05
E16	0.1125	8.67326E-05

Table S3. Normalizing weight matrix:

Sl.no	COF (X)	SWR (Y)
E1	0.03858165	3.75679E-05
E2	0.129741863	5.1312E-06
E3	0.023877666	0
E4	0.025841751	0
E5	0.035353535	4.21827E-06
E6	0.08493266	2.98491E-06
E7	0.029250842	5.04133E-05
E8	0.058403479	2.93875E-05
E9	0.063657407	1.34893E-07
E10	0.055667789	8.11651E-06
E11	0.02898569	2.14317E-06
E12	0.215025253	0.000172031
E13	0.076346801	6.33914E-05
E14	0.035079966	1.23944E-05
E15	0.072055976	1.00932E-05
E16	0.05625	4.33663E-05

Table S4. Positive and Negative ideal solution:

Responses	V^+	V^-
COF	0.02387	0.21502
SWR	0.00E+00	0.000172

Table S5. Separation matrix:

Sl.no	Si +	Si -
E1	0.014711698	0.347228376
E2	0.105871863	0.256068191
E3	7.66554E-06	0.361932375
E4	0.001971751	0.35996829
E5	0.011483536	0.350456505
E6	0.06106266	0.300877388
E7	0.005381078	0.356559179
E8	0.034533492	0.327406552
E9	0.039787407	0.322152638
E10	0.03179779	0.330142252
E11	0.005115691	0.35682435
E12	0.19115533	0.170784747
E13	0.05247684	0.309463218
E14	0.011209973	0.35073007
E15	0.048185977	0.313754065
E16	0.032380029	0.329560025

Table S6. Closeness coefficients value with rank order:

Sl.no	CCo	Rank
E1	0.959	7
E2	0.707	15
E3	1	1
E4	0.995	2
E5	0.968	6

E6	0.831	14
E7	0.985	4
E8	0.905	10
E9	0.89	11
E10	0.912	8
E11	0.986	3
E12	0.472	16
E13	0.855	13
E14	0.969	5
E15	0.867	12
E16	0.911	9

References:

1. B. B. Nayak,, S. S. Mahapatra Multi-response optimization of WEDM process parameters using the AHP and TOPSIS method. International Journal on Theoretical and Applied Research in Mechanical Engineering; Vol 2, (2013) 109-215.
2. Gadakh, V. S, Parametric Optimization of Wire Electrical Discharge Machining Using Topsis Method. Advances in Production Engineering & Management; Vol 7, No. 3, (2012). 157–164.
3. Rajesh K.B, B.C. Routara, Arun K.P, An approach for optimization the process parameter by using TOPSIS Method of Al–24%SiC metal matrix composite during EDM ; Volume 2, Issues 4–5, 2015, Pages 3116-3124.
4. X.LI, K. WANG, L. LIU, J. XIN, H. YANG, C.GAO, Application of the Entropy Weight and TOPSIS Method in Safety Evaluation of Coal Mines. Procedia Engineering, Vol.26, (2011). 2085 – 2091.
